# Assessment of the Implementation of Pharmacogenomic Testing in a Pediatric Tertiary Care Setting

**DOI:** 10.1001/jamanetworkopen.2021.10446

**Published:** 2021-05-26

**Authors:** Iris Cohn, Roozbeh Manshaei, Eriskay Liston, John B. A. Okello, Reem Khan, Meredith R. Curtis, Abby J. Krupski, Rebekah K. Jobling, Kelsey Kalbfleisch, Tara A. Paton, Miriam S. Reuter, Robin Z. Hayeems, Ruud H. J. Verstegen, Aaron Goldman, Raymond H. Kim, Shinya Ito

**Affiliations:** 1 Division of Clinical Pharmacology and Toxicology, Department of Paediatrics, The Hospital for Sick Children, University of Toronto, Toronto, Ontario, Canada; 2 Program in Translational Medicine, The Hospital for Sick Children, Toronto, Ontario, Canada; 3 Cardiac Genome Clinic, Ted Rogers Centre for Heart Research, The Hospital for Sick Children, Toronto, Ontario, Canada; 4 Division of Clinical and Metabolic Genetics, The Hospital for Sick Children, Toronto, Ontario, Canada; 5 Genome Diagnostics, Department of Pediatric Laboratory Medicine, The Hospital for Sick Children, Toronto, Ontario, Canada; 6 The Centre for Applied Genomics, The Hospital for Sick Children, Toronto, Ontario, Canada; 7 Canada’s Genomic Enterprise (CGEn), The Hospital for Sick Children, Toronto, Ontario, Canada; 8 Genetics and Genome Biology, Research Institute, The Hospital for Sick Children, Toronto, Ontario, Canada; 9 Program in Child Health Evaluative Sciences, The Hospital for Sick Children, Toronto, Ontario, Canada; 10 Institute of Health Policy Management and Evaluation, University of Toronto, Toronto, Ontario, Canada; 11 Division of Rheumatology, Department of Paediatrics, The Hospital for Sick Children, Toronto, Ontario, Canada; 12 Department of Paediatrics, University of Toronto, Toronto, Ontario, Canada; 13 DNALabs Canada Inc, Toronto, Ontario, Canada; 14 Fred A. Litwin Family Centre in Genetic Medicine, University Health Network, Department of Medicine, University of Toronto, Toronto, Ontario, Canada

## Abstract

**Importance:**

Pharmacogenomic (PGx) testing provides preemptive pharmacotherapeutic guidance regarding the lack of therapeutic benefit or adverse drug reactions of PGx targeted drugs. Pharmacogenomic information is of particular value among children with complex medical conditions who receive multiple medications and are at higher risk of developing adverse drug reactions.

**Objectives:**

To assess the implementation outcomes of a PGx testing program comprising both a point-of-care model that examined targeted drugs and a preemptive model informed by whole-genome sequencing that evaluated a broad range of drugs for potential therapy among children in a pediatric tertiary care setting.

**Design, Setting, and Participants:**

This cohort study was conducted at The Hospital for Sick Children in Toronto, Ontario, from January 2017 to September 2020. Pharmacogenomic analyses were performed among 172 children who were categorized into 2 groups: a point-of-care cohort and a preemptive cohort. The point-of-care cohort comprised 57 patients referred to the consultation clinic for planned therapy with PGx targeted drugs and/or for adverse drug reactions, including lack of therapeutic benefit, after the receipt of current or past medications. The preemptive cohort comprised 115 patients who received exploratory whole-genome sequencing–guided PGx testing for their heart conditions from the cardiac genome clinic at the Ted Rogers Centre for Heart Research.

**Exposures:**

Patients received PGx analysis of whole-genome sequencing data and/or multiplex genotyping of 6 pharmacogenes (*CYP2C19*, *CYP2C9*, *CYP2D6*, *CYP3A5*, *VKORC1*, and *TPMT*) that have established PGx clinical guidelines.

**Main Outcomes and Measures:**

The number of patients for whom PGx test results warranted deviation from standard dosing regimens.

**Results:**

A total of 172 children (mean [SD] age, 8.5 [5.6] years; 108 boys [62.8%]) were enrolled in the study. In the point-of-care cohort, a median of 2 target genes (range, 1-5 genes) were investigated per individual, with *CYP2C19* being the most frequently examined; genotypes in 21 of 57 children (36.8%) were incompatible with standard treatment regimens. As expected from population allelic frequencies, among the 115 children in the whole-genome sequencing–guided preemptive cohort, 92 children (80.0%) were recommended to receive nonstandard treatment regimens for potential drug therapies based on their 6-gene pharmacogenetic profile.

**Conclusions and Relevance:**

In this cohort study, among both the point-of-care and preemptive cohorts, the multiplex PGx testing program provided dosing recommendations that deviated from standard regimens at an overall rate that was similar to the population frequencies of relevant variants.

## Key Points

**Question:**

What is the clinical utility of a pharmacogenomic testing program that uses both point-of-care and preemptive approaches to assess potential responses to drugs in a pediatric tertiary care setting?

**Findings:**

In this cohort study of 172 pediatric patients, pharmacogenomic testing of 6 pharmacogenes (*CYP2D6*, *CYP2C9*, *CYP2C19*, *CYP3A5*, *TPMT*, and *VKORC1*) provided results that warranted deviation from standard treatment regimens in approximately 40% of patients in the point-of-care evaluation of targeted drugs and 80% of patients in the preemptive evaluation of a broader range of drugs for potential therapy.

**Meaning:**

The study’s findings suggest that a pharmacogenomic program using both point-of-care targeted drug–guided testing and preemptive whole-genome sequencing–guided testing enhances the knowledge necessary for patient care decision-making, providing informed rationales for drug selection and dosing options.

## Teaser

This cohort study assesses the implementation outcomes of a pharmacogenomic testing program comprising a point-of-care model evaluating targeted drugs and a preemptive model using whole-genome sequencing to evaluate potential drug therapies among children in a pediatric tertiary care setting.

## Introduction

Monitoring the efficacy and safety of medications prescribed to children is of substantial importance, especially in a tertiary care setting, in which patients typically have complex medical conditions that often require treatment with multiple medications. A study conducted among 1 million Canadian children found that only 20% of children accounted for 70% of all drug prescriptions.^1^ Those children were more likely to be diagnosed with chronic health conditions that required treatment in advanced care settings^1^ and were at higher risk of developing adverse drug reactions (ADRs).^2^ Multiple strategies are used to reduce the number of ADRs among children with complex conditions, and pharmacogenomics-guided drug therapy has emerged as one of the options.

Clinical pharmacogenomic (PGx) testing for drug-gene pairs with actionable practice guidelines has been widely recognized as a tool for medication selection and dosing that can be used to maximize the safety and welfare of patients.^3,4,5,6,7^ Previous surveillance studies among adults and children revealed that more than 90% of individuals have genetic variations that are associated with an increased risk of predictable and preventable harm from the receipt of medications that have pharmacogenomics-based guidelines.^8,9,10,11^ Despite the increasing number of pharmacogenomics implementation projects, the integration of PGx testing into real-time clinical care and decision-making has been challenging. These challenges have mainly been associated with implementation barriers, such as the paucity of regulated clinical PGx testing, limited literacy and comfort with regard to PGx testing among health care professionals, and the lack of integration of PGx data into the electronic health record.^12,13^

Two modes of PGx testing are available to identify the need for clinical evaluation regarding past, current, or upcoming drug therapy.^14^ The point-of-care model, also known as the reactive model, addresses only 1 or more targeted gene-drug combinations and is typically guided by clinical evaluation at the time of prescription or in response to an emerging or past ADR, including lack of therapeutic benefit. The preemptive PGx model is an active approach that addresses potential drug therapies by using genotyping strategies that involve the testing of multiple pharmacogenes regardless of an individual’s medication history. Although the use of a multiplex genotyping platform is standard when implementing the preemptive PGx approach, data from deep sequencing that were obtained for other purposes can also be used to extract PGx information.^11,15^ Given the diagnostic utility of whole-genome sequencing (WGS) in pediatric medicine, the preemptive WGS–guided PGx approach appears to be a reasonable option, particularly in tertiary care settings. This study assessed the implementation and clinical utility of a PGx testing program that used both a point-of-care targeted drug approach and a preemptive WGS-guided approach within a pediatric tertiary hospital.

## Methods

### Setting

This pilot cohort study was conducted in collaboration with the Division of Clinical Pharmacology and Toxicology at The Hospital for Sick Children, University of Toronto, and the Cardiac Genome Clinic at the Ted Rogers Centre for Heart Research.^16^ A pharmacogenomics clinic with a consultation program was established to advise health care professionals about pharmacogenomics-based therapeutic recommendations using annotated PGx guidelines. The study was approved by the research ethics board of The Hospital for Sick Children, and written informed consent was obtained on behalf of all participants. This study followed the Strengthening the Reporting of Observational Studies in Epidemiology (STROBE) reporting guideline for cohort studies.

### Participants

The study included 2 independent patient cohorts (point-of-care and preemptive) who were recruited through The Hospital for Sick Children.

#### Point-of-Care Cohort

The point-of-care cohort represented the targeted drug–guided approach and comprised 57 eligible patients (both outpatient and inpatient) who were enrolled by the clinical pharmacological consultation service between March 2017 and September 2020. By study design, patients were eligible for inclusion if they were designated to receive a drug and/or had developed an ADR or experienced lack of therapeutic benefit after receiving a drug (Table 1) that was annotated using PGx information detailed on the PharmGKB website^17^ and that included guidelines developed by the Clinical Pharmacogenetics Implementation Consortium, the Dutch Pharmacogenetics Working Group, and the US Food and Drug Administration (for labeling information). The medical condition for which pharmacotherapy was considered varied across patients in this cohort. Clinicians were able to contact the hospital’s clinical pharmacological consultation service with questions about patients who experienced unexpected drug responses and/or with requests for guidance on drug therapy. Patients receiving cancer and cancer-associated care were excluded from the study.

**Table 1.** Drug-Gene Interactions and Pharmacogenomic Variants Identified by Targeted Genotyping

Drug	Indication	Gene	SNV testing panel
Amoxapine, aripiprazole, atomoxetine, brexipiprazole, desipramine, fluvoxamine, haloperidol, nortriptyline, paroxetine, quinidine/dextrometorphan, perphenazine, pimozidine, protriptyline, tetrabenazine, thioridazine, valbenazine, venlafaxine, vortioxetine, and zuclopenthixol	Psychiatric disorders	*CYP2D6*	rs16947, rs1135840, rs35742686, rs3892097, rs5030655, rs5030867, rs5030865, rs5030656, rs1065852, rs201377835, rs5030864, rs5030862, rs5030865, rs72549357, rs28371706, 4133dupGTGCCCACT, rs72549353, rs72549354, rs59421388, rs28371735, rs28371725, CNV
Codeine, hydrocodone, oxycodone, and tramadol	Pain		
Ondansetron, metoclopramide, and tropisetron	Gastrointestinal disorders		
Flecainide and propafenone	Antiarrhythmics		
Metoprolol	β blocker		
Tolteridone	Bladder disorders		
Eliglustat	Gaucher disease		
Amitriptyline, clomipramine, doxepin, imipramine, and trimipramine	Psychiatric disorders	*CYP2D6* and *CYP2C19*	rs4244285, rs498689, rs28399504, rs56337013, rs72552267, rs72558186, rs41291556, rs12248560
Clopidogrel	Antiplatelet agent	*CYP2C19*	rs4244285, rs498689, rs28399504, rs56337013, rs72552267, rs72558186, rs41291556, rs12248560
Citalopram, clobazam, escitalopram, and sertraline	Psychiatric disorders		
Dexlansoprazole, lansoprazole, omeprazole, and pantoprazole	Gastrointestinal disorders		
Voriconazole	Infectious diseases		
Carisoprodol	Pain		
Warfarin	Anticoagulant	*VKORC1* and CYP2C9	rs9923231 (1639 G>A), rs1799853, rs1057910, rs56165452, rs28371686, rs9332131, rs7900194, rs28371685, rs9332239, rs72558187, rs72558190
Phenytoin, siponimod, and lesinurad	Neurologic disorders	*CYP2C9*	rs1799853, rs1057910, rs56165452, rs28371686, rs9332131, rs7900194, rs28371685, rs9332239, rs72558187, rs72558190
Celecoxib, flurbiprofen, ibuprofen, and meloxicam piroxicam	Pain		
Dronabinol	Antiemetic		
Azathioprine, mercaptopurine, and thioguanine	Autoimmune disorders and cancer	*TPMT*	rs1142345, rs1800460, rs1800462, rs1800584
Tacrolimus	Organ transplantation	*CYP3A5*	rs28365083, rs776746, rs10264272, rs41303343

Abbreviations: CNV, copy number variant; SNV, single nucleotide variant.

#### Preemptive Cohort

The preemptive cohort represented the WGS-guided PGx approach and comprised 115 children with cardiac disease who were recruited through the cardiac genome clinic between January 2017 and December 2019. This clinic was established by the Ted Rogers Centre for Heart Research to investigate genetic factors associated with an individual’s susceptibility to risk factors for heart failure and/or a diagnosis of heart failure. Patients in the cardiac genome clinic received exploratory WGS^16^ to identify potential genetic associations with their cardiac disease. As part of the analysis, PGx data were manually extracted from WGS data regardless of the presence or absence of planned or current receipt of a targeted drug. If a non-*1 haplotype was identified in 1 of 6 pharmacogenes (cytochrome P450, subfamily 2D, polypeptide 6 [*CYP2D6*; OMIM 124030); cytochrome P450, subfamily 2C, polypeptide 9 [*CYP2C9*; OMIM 601130], cytochrome P450, subfamily 2C, polypeptide 19 [*CYP2C19*; OMIM 124020]; cytochrome P450, subfamily 3Am polypeptide 5 [*CYP3A5*; OMIM 605325]; thiopurine S-methyltransferase [*TPMT*; OMIM 187680]; and vitamin K epoxide reductase complex, subunit 1 [*VKORC1*; OMIM 608547]) or a non-*3 haplotype was identified in cytochrome P450, subfamily 3A, polypeptide 5 (*CYP3A5*; OMIM 605325), the patient’s DNA sample was referred for further clinical validation via targeted genotyping at a laboratory with Clinical Laboratory Improvement Amendments certification.^16^

### Genotyping

For the point-of-care cohort, DNA was extracted using a buccal swab; for the preemptive cohort, banked DNA from whole blood was used to confirm the presence of PGx variants of interest. To infer haplotypes, all patients received genotyping of *CYP2C19*, *CYP2C9*, *CYP2D6*, *CYP3A5*, *TPMT*, and *VKORC1* using a mass spectrometer (MassARRAY Matrix-Assisted Laser Desorption Ionization–Time of Flight [MALDI-TOF]; Agena BioScience). Haplotype reports were automatically generated using MassARRAY TyperAnalyzer software, version 4.1.83, and iPLEX ADME PGx Pro software, version 3.99.105 (Agena BioScience), using the manufacturer’s standard protocols. Genotyping analysis was performed using a clinically validated commercial genetic testing company (Gene by Gene) that was accredited by the College of American Pathologists (CAP number: 7212851), the Clinical Laboratory Improvement Amendments (CLIA number: 45D1102202), the New York State Department of Health (NYSDOH number: 8512), the California Department of Public Health (CDPH number: COS00800486), and the American Association of Blood Banks (AABB number: 160062).

### Reporting

For each patient, 6 pharmacogenes were tested: *CYP2D6*, *CYP2C9*, *CYP2C19*, *CYP3A5*, *TPMT*, and *VKORC1*. Patients’ phenotype statuses could warrant a deviation in standard dosing based on the potential for variants to alter enzyme activity, as summarized in the drugs’ labeling information and established clinical practice guidelines (Table 1).^17^ All patients received a report that included genotyping results and phenotype interpretation (ie, metabolism status). Patients received counseling from a pharmacist and/or clinical pharmacologist regarding the implications of their PGx testing results. The patient’s health care team (Table 2) was informed to help guide clinical medication dosing, and the PGx report and consultation note were documented in the electronic health record of each patient.

**Table 2.** Clinical Service Referrals for Pharmacogenomic Consultation

Cohort^**a**^	Service	Patients referred, No. (N = 172)
Point-of-care	Cardiology	1
Point-of-care	Cardiac transplant	10
Point-of-care	Gastroenterology	12
Point-of-care	Genetics	3
Point-of-care	Hepatology	1
Point-of-care	Nephrology	1
Point-of-care	Neurology	10
Point-of-care	Psychiatry	18
Point-of-care	Rheumatology	1
Preemptive	Cardiac genome clinic	115

^a^ The point-of-care cohort comprised 57 patients recruited from the consultation clinic who received the targeted drug–guided approach. The preemptive cohort comprised 115 patients recruited from the cardiac genome clinic who received the whole-genome sequencing–guided approach.

### Statistical Analysis

To summarize PGx test outcomes, each patient was categorized based on the presence of pharmacogenomics-based recommendations that warranted deviation from standard therapeutic regimens. Descriptive statistics were used to summarize cohort characteristics.

## Results

A total of 172 children (mean [SD] age, 8.5 [5.6] years; 108 boys [62.8%]) were enrolled in the study. As shown in the Figure, 57 patients in the point-of-care cohort (mean [SD] age, 10.3 [5.5] years; 32 boys [56.1%]) were referred to the pharmacogenomics clinic for drug therapy guidance with regard to cardiovascular agents, proton pump inhibitors, and psychiatric medications. In the preemptive cohort, the WGS data of 115 children (mean [SD] age, 7.6 [5.4] years; 76 boys [66.1%]) were examined for PGx information within the 6 pharmacogenes. Overall, 126 participants (73.3%) were receiving care from cardiology services.

**Figure.**
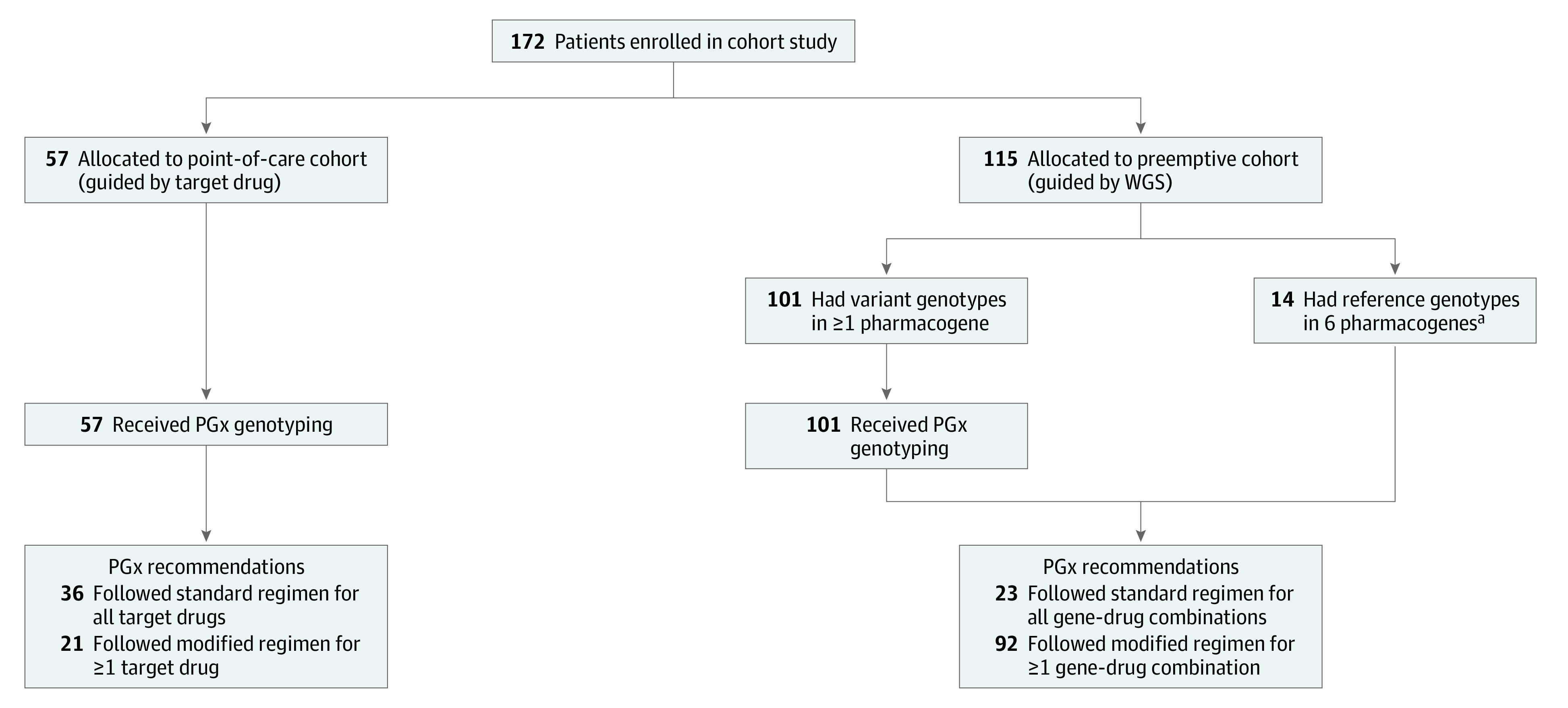
Patient Flowchart and Overview of Pharmacogenomic Data Integration PGx indicates pharmacogenomic; WGS, whole-genome sequencing. ^a^Non-*1 haplotype in *CYP2C9*, *CYP2C19*, *CYP2D6*, and *TPMT*; *VKORC1* 1639 G>A (GA variant in the presence of CYP2C9*1/*1 is defined as a reference); and non-*3 haplotype in *CYP3A5*.

### Point-of-Care Approach

In the point-of-care cohort, 24 of 57 children (42.1%) had initiated therapy with 1 or more medications listed in the pharmacogenomics guidelines before the consultation; 18 children (31.6%) received testing to inform upcoming therapy with one of the PGx targeted drugs, and 15 children (26.3%) received testing for both current and upcoming therapy with multiple targeted drugs. Overall, 39 children (68.4%) received testing for past index events of ADR or lack of therapeutic benefit, and 34 children (59.6%) received testing for upcoming drug therapy (eTable in the Supplement). In this cohort, the median number of the targeted genes investigated per individual was 2 (range, 1-5; mean [SD], 2.1 [1.3]). Gene *CYP2C19* was the most frequently examined (n = 52), followed by *CYP2D6* (n = 36), *CYP2C9* (n = 21), *CYP3A5* (n = 11), *VKORC1* (n = 9), and *TPMT* (n = 2) (eTable in the Supplement).

The test results for targeted drugs warranted treatment adjustments that deviated from the standard regimen in 21 children (36.8%), and the standard regimen was compatible with the genotypes of 36 children (63.2%) (Figure).

Because the PGx test was performed using the multiplex platform, children in the point-of-care cohort also received results for nontargeted drug-gene combinations of potential relevance. Coupled with the targeted drug results, these findings indicated that 29 children (50.9%) had at least 1 variant in the 6 pharmacogenes for which the drug and/or dosing recommendation differed from the standard regimen according to PGx guidelines (eTable in the Supplement).

### Preemptive Approach

In the preemptive cohort, 14 of 115 children (12.2%) exhibited the reference genotypes in all 6 pharmacogenes (Figure). Samples of DNA from the remaining 101 children (87.8%) were further tested using the multiplex PGx genotyping panel. The combined PGx results of the preemptive cohort indicated that a modified drug selection and/or dosing regimen for at least 1 drug-gene combination was recommended for 92 children (80.0%), while standard regimens were compatible with all tested genes in the remaining 23 children (20.0%) (eTable in the Supplement).

### Case Summaries

#### Warfarin

Pharmacogenomic test results indicated increased sensitivity to warfarin^18,19,20^ among 4 children who were actively receiving warfarin therapy. In 3 of these 4 patients, clinicians had difficulty determining the optimal therapeutic dose at initiation of warfarin therapy. In 1 instance, the international normalized ratio was found to be at supratherapeutic levels (ie, >5) after the initial standard dose of warfarin was administered, which put the child at an increased risk of bleeding.

#### Proton Pump Inhibitors

Five children who were already receiving proton pump inhibitors, such as pantoprazole, omeprazole, or lansoprazole, and experiencing little or no symptom relief were found to have rapid *CYP2C19* metabolism. This genotype has been associated with lower exposure to proton pump inhibitors, resulting in a lack of therapeutic benefit among patients receiving standard dosing. Thus, if PGx test results had been available a priori, a different dosing regimen and/or a medication not sensitive to the *CYP2C19* metabolizing enzyme would have been chosen.^21^ In another 4 patients, expected phenotypes of *CYP2C19* helped to further guide proton pump inhibitor dosing for long-term receipt because PGx results suggested that these patients had intermediate or low *CYP2C19* metabolism.^22,23^

#### Clopidogrel

Five children receiving clopidogrel, an antiplatelet medication, received genotyping and were found to have intermediate or low *CYP2C19* metabolism and a loss-of-function *CYP2C19* allele. Since clopidogrel therapy requires activation, mainly via *CYP2C19*, these children had an increased risk of experiencing a lack of therapeutic benefit.^24,25^ Three of the 5 children were precardiac transplant patients who experienced a lack of therapeutic benefit (thrombotic events) with clopidogrel therapy. The therapeutic regimen of all 5 patients was subsequently switched to an alternate drug (aspirin). Two children with increased *CYP2C19* activity (ie, rapid and ultrarapid metabolism status) had already received treatment with a standard dose of clopidogrel. These data provided an opportunity to inform patients and their clinicians about the increased risks of bleeding associated with the patients’ genotypes.^24,25,26^

#### Tacrolimus

Three heart transplantation recipients receiving tacrolimus were found to have *CYP3A5* expression. All 3 patients exhibited difficulty in achieving optimal dosing. Per Clinical Pharmacogenetics Implementation Consortium guidelines, these children would have benefited from an increased starting dose, as their increased metabolism interfered with achieving therapeutic levels of tacrolimus based on standard dosing guidelines.^27,28^

#### Ondansetron

One patient had a duplication of *CYP2D6* functioning alleles and was therefore considered to have ultrarapid *CYP2D6* metabolism. This genotype is associated with increased metabolism of ondansetron,^29^ and, as expected, impaired therapeutic response to ondansetron was observed.

#### Selective Serotonin Reuptake Inhibitors

The point-of-care group included children and adolescents who either experienced an impaired therapeutic response to antidepressant medications or received evaluations for potential psychiatric treatment with drugs included in PGx guidelines (Table 1). In 13 of these patients, expected *CYP2D6* and *CYP2C19* enzyme activities warranted the dosing and/or drug choice of selective serotonin reuptake inhibitors and tricyclic antidepressant medications.^30,31,32,33^ The use of pharmacogenomic data enhanced previous information on pharmacokinetic profiles to guide therapeutic management, providing the rationale for dose changes and an increased safety profile among 9 patients.^30,31,32,33^ In 1 patient, reduced *CYP2C19* metabolism was consistent with excessive weight gain from the previous receipt of escitalopram therapy.^31^

## Discussion

 This cohort study describes the process outcomes^14^ of the clinical implementation of a pharmacogenomics program in a pediatric tertiary hospital among both outpatients and inpatients. In the point-of-care cohort, approximately 40% of test results warranted modification of standard dosing regimens. In the preemptive cohort, 80% of test results supported deviation from standard dosing regimens with potential drugs based on at least 1 of the 6 pharmacogenes (Figure). These frequencies of 40% to 80% for nonstandard dosing recommendations are consistent with the combined event rates of the respective genes involved.^8^

Owing to its potential to estimate an individual patient’s treatment response, PGx testing is becoming one of the pillars of individualized precision health care.^34^ However, data on the implementation outcomes of such a program among a pediatric population are scarce.^34,35,36^ The current results focused on deviation from standard regimens, but PGx results warranting the use of a standard regimen are equally important in clinical pharmacotherapeutic decision-making. The term *actionable phenotypes* is misleading if used only to describe phenotype results warranting deviation from standard regimens because results supporting both modified and standard regimens are actionable. Overall, the study’s results suggest that a PGx testing program in a tertiary care setting provides important information to guide patient care decisions for children with complex medical problems, and these findings are comparable with those of previous studies.^33,34,35,36,37,38,39^

Overall, 73.3% of study participants were receiving care from cardiology services (Table 2), which explains the common receipt of medications such as clopidogrel, tacrolimus, and warfarin in the patient cohorts. Although limited by our patient selection, the results of this pilot study are consistent with those of previous studies performed among adult and pediatric populations, which reported the clinical benefits of identifying patients with an increased risk of experiencing ADRs after receiving standard dosing of high-risk medications.^24,40,41,42^

The importance of using PGx testing to advance improvements in heart transplant clinical care and patient outcomes has been debated.^28,43^ To our knowledge, the present study provides the first comprehensive set of PGx data for pediatric heart transplantation. We informed clinicians about PGx variants present in 8 cardiac transplant candidates and 4 heart transplant recipients, which may have helped to prevent or explain treatment-associated adverse events and resulted in modified dosing regimens for 75% of those children. For example, 3 of the 4 heart transplant recipients experienced variability in tacrolimus drug concentrations that increased rejection risk owing to underdosing based on their *CYP3A5* enzyme expression.^27,28^ Clopidogrel, a platelet aggregation inhibitor, is often used as an additive agent to prevent pretransplantation thrombotic events among children with left ventricular assistive devices, such as the Berlin Heart EXCOR (Berlin Heart Group).^25^ However, 4 children experienced a thromboembolic event while receiving clopidogrel, and 3 of those children had reduced *CYP2C19* activity. Warfarin, an anticoagulant, is commonly prescribed in pediatric populations, mainly for the treatment of thromboembolic disorders, as prophylaxis for heart valve replacement, after the receipt cavopulmonary shunts, and before Fontan completion surgery among patients with complex congenital heart disease.^19,20^ In children, a large proportion of the variability in warfarin dose requirements may be explained by the variants in *VKORC1* and *CYP2C9*, as observed in 3 of the children in our study who were considered warfarin-sensitive.^19,21,42^ Further studies are needed to assess the prospective value of genotype-guided dosing at initiation of and during warfarin therapy in the pediatric population.

 Testing via WGS has become increasingly common among children with undiagnosed complex medical conditions.^44,45^ In this study, WGS was incorporated into the PGx analysis framework.^11^ As articulated by Relling and Evans,^39^ the debate about PGx testing has shifted from a scheme of “whether to order a genetic test”^39^^(p347)^ to that of “how existing genetic test results can and should be used to influence prescribing decisions.”^39^^(p347)^

The present study examined the implementation outcomes and benefits of PGx testing in 2 different cohorts, and the findings indicated that clinically useful information can be derived from both reactive and prospective testing. However, as the field of pharmacogenomics continues to expand and testing becomes more widely available, we propose that PGx testing and/or analysis (in the context of genome sequencing conducted for other medical reasons) be performed in a preemptive manner as often as possible. Although reactive testing results can help to explain previous adverse effects and/or therapeutic shortcomings, they often occur in a context in which a patient is harmed to some extent. Pharmacogenomic testing conducted in a preemptive manner would likely prevent these clinical scenarios from occurring and therefore provide patient care that is safer and more efficient while curtailing substantial expenses, as reported in a recent cost-effectiveness analysis of preemptive pharmacogenetic tests.^46^

### Limitations

This study has limitations. The comprehensive clinical utility of PGx testing warrants further analysis, given that each drug-gene combination was not explored separately in the point-of-care cohort because of sample size limitations. Therefore, it was not possible to analyze the diagnostic value of PGx tests for assessing past or ongoing ADRs or lack of therapeutic benefit specific to each gene-drug pair. Multi-institutional collaborative efforts are needed to examine these questions.

Notably, pharmacodynamic factors, including receptor and transporter variations in neurotransmitters, have been identified as important factors associated with the therapeutic benefits of antidepressant, antipsychotic, stimulant, and antiadrenergic medications.^33^ These pharmacodynamic factors will eventually need to be integrated into PGx testing, and their variations are currently being investigated for use as psychopharmacologic interventions among children and adults.^33^ Further research is warranted to increase the clinical utility of PGx testing among children and adolescents with mental health conditions.

## Conclusions

This study’s findings add to the increasing body of evidence indicating that providing a profile of 6 pharmacogenes (*CYP2D6*, *CYP2C9, CYP2C19*, *CYP3A5*, *TPMT*, and *VKORC1*) and implementing a PGx consultation program are of clinical benefit for children who require treatment with certain medications. The current implementation program suggests that PGx data has real-world value in routine clinical care among a pediatric cohort. Because the use of clinical genome sequencing is expected to further increase,^37,47,48^ this study’s findings may be useful to the increasing effort to advance the application and understanding of PGx data in routine pediatric clinical care.

## Notes

Supplement. **eTable.** Patient Data by Study Group for Children With Past, Current, and Imminent Drug Therapy
