# Supplementary material for: Assessment of the Implementation of Pharmacogenomic Testing in a Pediatric Tertiary Care Setting
Source: JAMA Netw Open. 2021 May 26;4(5):e2110446. doi: 10.1001/jamanetworkopen.2021.10446 (PMC8155824; doi:10.1001/jamanetworkopen.2021.10446)
Supplement: Supplement. — eTable. Patient Data by Study Group for Children With Past, Current, and Imminent Drug Therapy [file jamanetwopen-e2110446-s001.pdf]

Supplementary Online Content

Cohn I, Manshaei R, Liston E, et al. Assessment of the implementation of pharmacogenomic testing in a pediatric tertiary care setting. *JAMA Netw Open*. 2021;4(5):e2110446. doi:10.1001/jamanetworkopen.2021.10446

eTable. Patient Data by Study Group for Children With Past, Current, and Imminent Drug Therapy

This supplementary material has been provided by the authors to give readers additional information about their work.

© 2021 Cohn I et al. *JAMA Network Open*.

eTable. Patient Data by Study Group for Children With Past, Current, and Imminent Drug Therapy

Patient	Number of inquired genes (reactive)	Number of inquired genes (pre-emptive)	Inquired drugs	PGx test reason	PGx use
CC1	2: CYP2C9 (IM), CYP2C19 (IM)	0	(cannabinoids), ibuprofen, omeprazole	partial response to PPI use	current/past drug therapy
CC2	0	1: CYP2C9 (IM)	not on PGx drugs	experienced severe hypersensitivity reaction on carbamazepine; use of other anticonvulsants	imminent drug therapy
CC3	0	1: TPMT (NM)	not on PGx drugs	planned use of azathioprine	imminent drug therapy
CC4	0	3: CYP2C9 (IM), VKORC1 (normal function), CYP2C19 (NM)	not on PGx drugs	WES sequencing; PGx validation- congenital heart disease	imminent drug therapy
CC5	1: CYP2C19 NM	0	rabeprazole	unsuccessful PPI use	current/past drug therapy
CC6	0	1: CYP2C9 (IM)	not on PGx drugs	planned anticonvulsant therapy	imminent drug therapy
CC7	1: CYP2C19 IM	0	omeprazole	unsuccessful PPI use	current/past drug therapy
CC8	0	1: CYP2C9 (IM)	not on PGx drugs	experienced hypersensitivity reaction on carbamazepine; use of other anticonvulsants	imminent drug therapy
CC9	0	1: CYP2C9 (NM)	not on PGx drugs	experienced hypersensitivity reaction on carbamazepine; use of other anticonvulsants	imminent drug therapy
CC10	1: CYP2C19 (IM)	0	rabeprazole	partial response on high doses of PPI's	current/past drug therapy
CC11	3: CYP3A5 (IM), CYP2D6 (NM), CYP2C19 (NM)	0	flecainide, omeprazole, tacrolimus	cardiac transplant recipient; difficulties achieving optimal dosing with tacrolimus	current/past drug therapy

© 2021 Cohn I et al. *JAMA Network Open*.

CC12	0	2: CYP2C19 (PM), CYP2D6 (NM)	oxcarbazepine	planned SSRI/ psychopharmaca use	imminent drug therapy
CC13	0	1: CYP2C9 (NM)	not on PGx drugs	experienced hypersensitivity reaction on carbamazepine; use of other anticonvulsants (at the time we tested HLA-A*3101)	imminent drug therapy
CC14	2: CYP2C19 (PM), CYP2D6 (NM)	0	ondansetron, pantoprazole	partial response on high doses of PPI's	current/past drug therapy
CC15	0	1: CYP2D6 (UM)	not on PGx drugs	Antley- Bixler syndrome- CYP2D6 status	imminent drug therapy
CC16	0	1: CYP2C9 (NM)	not on PGx drugs	experienced hypersensitivity reaction on carbamazepine; use of other anticonvulsants	imminent drug therapy
CC17	3: CYP2C9 (PM), CYP2C19 (NM), CYP2D6 (NM)	0	naproxen, sertraline	serotonin syndrome while on SSRI's (fluoxetine, sertraline)	current/past drug therapy
CC18	1: CYP2C19 (NM)	4: CYP3A5 (PM), CYP2D6 (NM), CYP2C9 (NM), VKORC1 (reduced function)	clopidogrel, lansoprazole	cardiac transplant candidate; may be given ondansetron, tacrolimus, warfarin	imminent drug therapy + current/past drug therapy
CC19	2: CYP2C19 (IM), CYP2D6 (NM)	3: CYP3A5 (PM), CYP2C9 (NM), VKORC1 (reduced function)	metoprolol, omeprazole	cardiac transplant candidate; may be given clopidogrel, ondansetron, tacrolimus, warfarin	imminent drug therapy + current/past drug therapy
CC20	2: CYP2C19 (IM), CYP2D6 (NM)	3: CYP3A5 (PM), CYP2C9 (IM), VKORC1 (reduced function)	diazepam, metoprolol, omeprazole	cardiac transplant candidate; may be given clopidogrel, ondansetron, tacrolimus, warfarin	imminent drug therapy + current/past drug therapy
CC21	2: CYP2C19 (IM), CYP3A5 (PM)	0	lansoprazole, tacrolimus	cardiac transplant recipient; expericenced GI bleed and thrombotic event on pantoprazole, clopidogrel	current/past drug therapy

© 2021 Cohn I et al. *JAMA Network Open*.

CC22	1: CYP2C19 (NM)	0	omeprazole	unsuccessful PPI use (omeprazole)	current/past drug therapy
CC23	1: CYP2C19 (NM)	0	omeprazole	unsuccessful PPI use (omeprazole)	current/past drug therapy
CC24	2: CYP2D6 (NM), CYP2C19 (NM)	0	aripiprazole	partial response to SSRI (sertraline)	current/past drug therapy
CC25	0	1: CYP2C9 (NM)	oxcarbazepine	experienced severe hypersensitivity reaction on carbamazepine; use of other anticonvulsants	imminent drug therapy
CC26	1: CYP2C19 (NM)	0	omeprazole	non responsive to PPI use	current/past drug therapy
CC27	0	2: CYP2D6 (NM), CYP2C19 (NM)	not on PGx drugs	planned SSRI/ psychopharmaca use	imminent drug therapy
CC28	2: CYP2C19 (NM), CYP2D6 (IM)	0	none	nose bleeds while on paroxetine	current/past drug therapy
CC29	0	2: CYP2D6 (NM), CYP2C19 (IM)	none	planned SSRI/ psychopharmaca use	imminent drug therapy
CC30	1: CYP2C19 (IM)	0	pantoprazole	unsuccessful PPI use	current/past drug therapy
CC31	2: CYP2D6 (NM), CYP2C19 (NM)	2: CYP2D6 (NM), CYP2C19 (NM)	not on PGx drugs	non response to SSRI/ psychopharmaca	imminent drug therapy + current/past drug therapy
CC32	2: CYP2D6 (NM), CYP2C19 (IM)	0	clopidogrel, metoprolol	thrombotic events, started on clopidogrel	current/past drug therapy
CC33	1: CYP2D6 (IM)	1: CYP2C19 (IM)	risperidone	partial response to SSRI/ future use of SSRI	imminent drug therapy + current/past drug therapy
CC34	2: CYP2D6 (NM), CYP2C19 (IM)	0	risperidone, sertraline	partial response to SSRI	current/past drug therapy
CC35	2: CYP2D6 (NM), CYP2C19 (NM)	0	amitriptyline, ondansetron, pantoprazole,risperidone	unsuccessful PPI use	current/past drug therapy
CC36	1: CYP2C9 (NM)	1: CYP2C9 (NM)	not on PGx drugs	behavioral changes to antiepileptics	imminent drug therapy +

© 2021 Cohn I et al. *JAMA Network Open*.

					current/past drug therapy
CC37	2: CYP2D6 (NM), CYP2C19 (NM)	0	fluvoxamine	unresponsive to SSRI	current/past drug therapy
CC38	1: CYP2C19 (IM)	0	escitalopram	excessive weight gain with escitalopram	current/past drug therapy
CC39	2: CYP2C19 (PM), CYP2D6 (NM)	0	clopidogrel, ondansetron	cerebellar thromobotic events while on clopidogrel	current/past drug therapy
CC40	4: CYP2C19 (NM), CYP2D6 (IM), CYP2C9 (NM), VKORC1 (reduced function)	2: CYP2C19 (NM), CYP3A5 (PM)	lansoprazole, ondansetron, warfarin	cardiac transplant candidate; may be given clopidogrel and tacrolimus	imminent drug therapy + current/past drug therapy
CC41	1: CYP2D6 (IM)	2: CYP2C19 (IM), CYP2D6 (IM)	aripiprazole, fluvoxamine	partial response to SSRI/ psychopharmaca	imminent drug therapy + current/past drug therapy
CC42	1: CYP2C19 (NM)	2: CYP2C19 (NM), CYP2D6 (NM)	sertraline	partial responsive to SSRI	imminent drug therapy + current/past drug therapy
CC43	4: CYP2C19 (NM), CYP2D6 (NM), CYP2C9 (NM), VKORC1 (normal function)	3: CYP2C19 (NM), CYP3A5 (IM), CYP2D6 (NM)	warfarin, lansoprazole, ibuprofen	cardiac transplant candidate; may be given clopidogrel, ondansetron, tacrolimus	imminent drug therapy + current/past drug therapy
CC44	0	2: CYP2C19 (RM), CYP2D6 (NM)	not on PGx drugs	planned SSRI/ psychopharmaca use	imminent drug therapy
CC45	3: CYP2D6 (NM), CYP2C9 (NM), VKORC1 (normal function)	2: CYP2C19 (IM), CYP3A5 (PM)	ondansetron, warfarin	cardiac transplant candidate; may be given clopidogrel, tacrolimus	imminent drug therapy + current/past drug therapy

© 2021 Cohn I et al. *JAMA Network Open*.

CC46	2: CYP2C9 (IM), CYP2C19 (NM)	2: CYP2D6 (NM), CYP2C19 (NM)	lansoprazole, naproxen	planned SSRI/ psychopharmaca use	imminent drug therapy + current/past drug therapy
CC47	1: CYP2D6 (NM)	2: TPMT (NM), CYP3A5 (IM)	metoprolol	planned use of azathioprine, tacrolimus	imminent drug therapy + current/past drug therapy
CC48	1: CYP2C19 (IM)	1: CYP2C19 (IM)	lansoprazole	portal vein thrombosis; imminent clopidogrel use	imminent drug therapy + current/past drug therapy
CC49	1: CYP2C19 (NM)	0	diazepam, pantoprazole	unsuccessful PPI use	current/past drug therapy
CC50	1: CYP2C19 (IM)	4: CYP2D6 (UM), CYP2C9 (NM), CYP3A5 (PM), VKORC1 (normal function)	clopidogrel	cardiac transplant candidate; may be given tacrolimus, warfarin, ondansetron	imminent drug therapy + current/past drug therapy
CC51	0	5: CYP2D6 (NM), CYP2C19 (NM), CYP2C9 (NM), CYP3A5 (PM), VKORC1 (normal function)	none	cardiac transplant candidate; may be given clopidogrel, ondansetron, tacrolimus, warfarin	imminent drug therapy
CC52	0	2: CYP2C19 (PM), CYP2D6 (IM)	none	planned SSRI/ psychopharmaca use	imminent drug therapy
CC53	1: CYP2C19 (NM)	0	omeprazole	unsuccessful PPI use- dose adjusted back to normal range	current/past drug therapy
CC54	2: CYP2C19 (RM), CYP2D6 (IM)	0	rabeprazole, tetrabenazine	unsuccessful PPI use	current/past drug therapy
CC55	0	2: CYP2C19 (RM), CYP2D6 (NM)	not on PGx drugs	planned SSRI/ psychopharmaca use	imminent drug therapy
CC56	0	2: CYP2D6 (NM), CYP2C19 (NM)	not on PGx drugs	planned SSRI/ psychopharmaca use	imminent drug therapy
CC57	1: CYP2C19 (IM)	0	clopidogrel	started on clopidogrel; experienced 2nd stroke	current/past drug therapy
CGC11	1: CYP3A5 (NM)	0	tacrolimus	cardiac transplant recipient; difficulties achieving optimal dosing with tacrolimus	current/past drug therapy
CGC15	1: CYP2C19 (RM)	0	clopidogrel	cardiac disease	current/past drug therapy

© 2021 Cohn I et al. *JAMA Network Open*.

CGC17	2: CYP2C9 (NM), VKORC1 (normal function)	0	warfarin	cardiac disease	current/past drug therapy
CGC19	2: CYP2D6 (IM), CYP2C19 (IM)	0	aripiprazole, atomoxetine, lansoprazole	cardiac disease	current/past drug therapy
CGC20	2: CYP2C19 (RM), CYP2C9 (NM)	0	clopidogrel, ibuprofen, omeprazole	non responsive to PPI use	current/past drug therapy
CGC30	1: CYP2C19 (NM)	2: CYP2C9 (IM), VKORC1 (reduced function)	lansoprazole	planned warfarin therapy	imminent drug therapy + current/past drug therapy
CGC31	1: CYP2C19 (RM)	0	none	partial response to omeprazole	current/past drug therapy
CGC34	1: CYP2C19 (RM)	1: CYP2C19 (RM)	omeprazole	planned to receive clopidogrel, but will be changed to aspirin	imminent drug therapy + current/past drug therapy
CGC35	1: CYP2C19 (UM)	0	clopidogrel	experienced bleeding incident on clopidogrel	current/past drug therapy
CGC38	2: CYP2C9 (IM), VKORC1 (reduced function)	0	warfarin	cardiac disease	current/past drug therapy
CGC43	1: CYP2C19 (IM)	0	omeprazole	cardiac disease	current/past drug therapy
CGC44	1: CYP2C19 (RM)	0	none	unresponsive to PPI use	current/past drug therapy
CGC45	0	5: CYP2C19 (PM), CYP2C9 (NM),CYP2D6 (NM), CYP3A5 (IM), VKORC1 (normal function)	not on PGx drugs	cardiac disease and liver disease; may be given clopidogrel, ondansetron, tacrolimus, warfarin	imminent drug therapy
CGC46	1: CYP3A5 (IM)	0	tacrolimus	cardiac transplant recipient; difficulties achieving optimal dosing with tacrolimus	current/past drug therapy

© 2021 Cohn I et al. *JAMA Network Open*.

CGC50	2: CYP2C9 (IM), VKORC1 (poor function)	1: CYP2C19 (UM)	not on PGx drugs	cardiac disease; may be given clopidogrel, was on warfarin perviously	imminent drug therapy + current/past drug therapy
CGC53	1: CYP2C19 (RM)	0	lansoprazole	partial response to lansoprazole	current/past drug therapy
CGC54	1: VKORC1 (poor function)	0	warfarin	INR tends to become supratherapeutic	current/past drug therapy
CGC55	0	3: CYP2C19 (NM), CYP2C9 (IM), VKORC1 (poor function)	not on PGx drugs	cardiac disease; may be given clopidogrel, warfarin	imminent drug therapy
CGC56	1: VKORC1 (poor function)	0	not on PGx drugs	warfarin use in the past; difficulties adjusting doses	current/past drug therapy
CGC57	1: CYP2D6 (UM)	0	none	side effects while on codeine (difficulties waking up)	current/past drug therapy
CGC59	1: CYP2D6 (UM)	0	not on PGx drugs	partial response to ondansetron	current/past drug therapy
CGC62	0	3: CYP2C19 (IM), CYP2C9 (NM), VKORC1 (poor function)	none	cardiac disease; may be given clopidogrel, warfarin	imminent drug therapy
CGC65	2: CYP2C9 (PM), VKORC1 (poor function)	0	warfarin	supratherapeutic INR 8 at the beginning of therapy	current/past drug therapy
CGC68	0	3: CYP2C19 (IM), CYP2C9 (NM), VKORC1 (reduced function)	not on PGx drugs	cardiac disease; may be given clopidogrel, warfarin	imminent drug therapy
CGC69	1: CYP2C19 (RM)	0	not on PGx drugs	unresponsive to PPI use	current/past drug therapy
CGC70	0	3: CYP2C19 (IM), CYP2C9 (NM), VKORC1 (normal function)	not on PGx drugs	cardiac disease; may be given clopidogrel, warfarin	imminent drug therapy
CGC72	2: CYP2C9 (NM), VKORC1 (normal function)	1: CYP2C19 (IM)	warfarin	congenital heart disease; hx of anticoagulation, GERD	imminent drug therapy + current/past drug therapy
CGC74	1: CYP2C9 (IM)	0	ibuprofen	cardiac disease	current/past drug therapy

© 2021 Cohn I et al. *JAMA Network Open*.

CGC76	0	2: CYP2C9 (PM), VKORC1 (reduced function)	not on PGx drugs	may be given warfarin in the near future	imminent drug therapy
CGC80	1: CYP2C19 (RM)	0	not on PGx drugs	unresponsive to PPI (lansoprazole) use	current/past drug therapy
CGC90	1: CYP2C19 NM	0	lansoprazole	cardiac disease	current/past drug therapy
CGC93	0	3: CYP2C19 (NM), CYP2C9 (NM), VKORC1 (poor function)	not on PGx drugs	cardiac disease; may be given clopidogrel, warfarin	imminent drug therapy
CGG94	2: CYP2C9 (NM), VKORC1 (normal function)	0	warfarin	cardiac disease	current/past drug therapy
CGC95	1: CYP2C19 (RM)	0	none	unresponsive to PPI (omeprazole) use	current/past drug therapy
CGC100	0	3: CYP2C19 (NM), CYP2C9 (NM), VKORC1 (reduced function)	not on PGx drugs	cardiac disease; may be given clopidogrel, warfarin	imminent drug therapy
CGC109	3: CYP2D6 (NM), CYP2C9 (NM), VKORC1 (reduced function)	1: CYP2C19 (RM)	flecainide, warfarin	cardiac disease	current/past drug therapy
CGC111	1: CYP2C19 (RM)	0	omeprazole	cardiac disease	current/past drug therapy
CGC112	1: CYP2C9 (IM)	0	ibuprofen	cardiac disease	current/past drug therapy

Abbreviations: CC, consultation cohort; CGC, cardiac genome clinic; IM, intermediate metabolizer; NM, normal metabolizer; PM, poor metabolizer; RM, rapid metabolizer; UM, ultrarapid metabolizer

© 2021 Cohn I et al. *JAMA Network Open*.
